# Seeking professional help for sleep-related complaints

**DOI:** 10.3389/fpubh.2024.1430574

**Published:** 2024-12-05

**Authors:** Lisa Rauch, Torsten Schneider, Claus Wendt

**Affiliations:** Department of Social Science, University of Siegen, Siegen, Germany

**Keywords:** sleep, sleep complaints, mental health, stigmatization, self-perceived cause, CBT-I, medication

## Abstract

**Introduction:**

Sleep-related complaints affect a significant proportion of the adult population in many societies. Despite the prevalence of symptoms and potential secondary and comorbid conditions, the utilization of professional help is quite low. The underlying reasons for this phenomenon have yet to be adequately investigated. To address this gap, we expand the conventional explanatory models to encompass the perceived cause as an explanatory factor. This is aimed to contribute to a better understanding of the help-seeking behavior and to create the basis for possible measures.

**Materials and methods:**

The empirical basis of the study is a quantitative data set collected in Germany in 2015 comprising 3,000 individuals between the ages of 40 and 75. Of these individuals, 761 reported experiencing sleep-related complaints. In our logistic regression, the binary dependent variable is whether professional help has already been sought. In addition to the factors included in the behavioral model of health service use, we utilize the perceived cause as a predictor, categorizing the variable as follows: exclusively mental, partially mental, and not mental.

**Results:**

Our analysis shows that individuals who attributed their sleep disturbances solely to their psyche were significantly less likely to seek professional help (AME: −0.158**). These differences remain significant after controlling for the severity of the complaints, their duration, and other covariates (AME: −0.117**). The data also suggest that this group is more likely to seek self-help through medication.

**Discussion:**

The results indicate the importance of focusing healthcare systems and public health policy on sleep-related complaints and their management. The aim is not only to alleviate sleep complaints, but also to reduce the risk of secondary diseases and to identify possible comorbidities. Additionally, it is a factor in reducing safety risks for the general public. Therefore, various measures should be implemented, including destigmatizing, improving health literacy, a more comprehensive offer of cognitive-behavioral therapy (CBT-I), and a more thorough examinations by physicians.

## 1 Introduction

Sleep-related complaints are prevalent phenomenon in modern societies. In many countries, approximately one-third of adults reporting symptoms (1–3). Symptoms can be dissatisfaction with quality or quantity of sleep, daytime sleepiness, and problems in initiating or maintaining sleep (4). Several studies have identified negative effects of sleep deprivation, such as an increased risk of memory problems, coronary heart disease, type 2 diabetes, cognitive impairment and accidents (5–9). According to these and other studies, sleep complaints reduce individual and societal health and well-being, increase healthcare costs, and have a negative impact on productivity (10–12).

Despite the high individual and societal costs, studies show that the step of seeking professional help for sleep complaints is very rarely taken. In Canada, for instance, the use of professional help for sleep-related complaints is as low as 13% (2). One possible explanation for this behavior could be that sleep complaints often have a psychological cause (13, 14). It is argued that the rate of help-seeking in cases of mental health issues is relatively low especially for the older adults (15–17). This, in turn, could be related to the stigmatization that still surrounds mental health issues (18).

A number of studies have identified stigmatization as a major barrier to healthcare utilization (19–21). While stigmatization and healthcare utilization in general is well studied, less is known about why people with sleep-related complaints rarely seek professional help. We hypothesize that underutilization in case of sleep complaints are related to a perceived stigmatization of mental health problems (22). In alignment with the findings of Stolzenburg et al. (23), which indicate that individuals with an untreated depressive syndrome who do not have a biomedical explanation for their symptoms tend to have a lower utilization rate, we formulate the following research question in relation to sleep complaints: Are individuals experiencing sleep-related complaints less likely to seek professional help if they perceive the underlying cause to be psychological?.

To answer this question, we use a quantitative data set that includes middle-aged and older adults with sleep complaints (24). This group is distinguished by a high prevalence of sleep disorders and a notable reluctance to utilize services for mental health impairments (25, 26). The objective of this study is to enhance understanding of the low utilization of professional help for sleep complaints and to highlight potential strategies that could facilitate an increase in such utilization. This could contribute to more appropriate treatment and an enhanced quality of life for those affected, as well as increased productivity for society as a whole and a reduction in the risk of accidents.

### 1.1 Help-seeking theory

According to the theory of help-seeking in case of sickness (27), the use of professional help is the final stage of the help-seeking process. Whether these steps are taken and how long it takes to take the single steps depends on many different factors. Andersen’s Behavioral Model of Health Services is considered a well-established analytical basis for explaining help-seeking behavior (28–30). Basically, it distinguishes between three factors that influence the decision to seek help. These are predisposing factors, enabling resources, and need. Predisposing factors are individual characteristics that exist before or independently of the occurrence of a particular episode of illness. These indirect factors are primarily demographic and socio structural such as gender, age, education, occupation, and ethnicity. In addition, Andersen includes general (health) attitudes and health awareness. Even if there is a belief that a physician’s treatment could help, there are preconditions for seeking care in the first place. For example, there may be some costs associated with seeking care, which requires the availability of financial resources. Enabling resources include above all the financial aspects or the type of health insurance. The accessibility of healthcare providers may also play a role, for example, if the density of healthcare providers is lower in rural than in urban areas. The most direct influence on decision making is the disease itself (need). In order to have a need, the disease must first be perceived, and its extent and severity determined. Sufferers can assess this, for example, by the extent of their daily impairment. The exact description of how we operationalized the factors in our analysis is given in the Methods section. Before, we discuss in more detail what additional role stigmatization might play in the context of seeking professional help.

### 1.2 Stigmatization

Goffman describes stigmatization as the process of assigning negative attributes to individuals or groups, thereby discrediting them and denying them social acceptance (31). Today, stigma is used as a sociological and social psychological concept that consists of three components: stereotypes, collective prejudice, and discrimination (32). People are thus devalued and discriminated against on the basis of one or more characteristics. People who are stigmatized usually experience poorer health and quality of life, which sometimes extends to their social environment (33, 34). Health-related stigma accounts for a large part of the research in this area that shows a broad evidence of stigma toward persons with mental illnesses such as schizophrenia and depression (35, 36). A decisive aspect is so-called self-stigmatization. This is a form of stigma in which individuals identify characteristics of themselves that are stigmatized or feel that they belong to a stigmatized group (37). These individuals assume that they do not deserve the same esteem as others and are afraid of being devalued by others, which leads to passive behavior (38). Therefore, stigmatization is considered a key barrier to seeking healthcare services (19–21). It does not only lead to lower utilization but is often accompanied by an increase in health impairments (39, 40). Since sleep-related complaints often have or are associated with a psychological cause (13, 14), this could be a central explanatory factor for low utilization of professional help by certain risk groups.

### 1.3 Earlier studies

A large number of the factors have been empirically verified in previous studies on help-seeking behavior. Regarding socioeconomic factors, women, younger adults as well as people with higher education are more likely to use the healthcare system (41–43). A crucial factor for help-seeking is the severity of the symptoms. The more severe the symptoms, the more likely it is visiting a professional (44). In the context of attitudes, people who are more likely to agree with self-help beliefs are less likely to use professional help (45).

As already mentioned, it also seems to play a role whether the suffering is perceived as something mental or more physical. An American study revealed that less than 30% of those who met the criteria for a psychiatric diagnosis according to the DSM-IV did actually use professional help (17). Those who know mental health professionals and those who communicate their complaints more openly in turn tend to use professional help significantly more (46). It has been shown that women generally exhibit a more positive attitude toward seeking professional help in case of mental illness and have higher trust in healthcare providers than men (47). Having trust in physicians was found to be a positive factor for seeking professional help in case of mental problems (48). However, when depression is accompanied by physical pain, people are more likely to seek help (49). This, in turn, supports our thesis that help seeking is more likely if there is not solely a mental health problem. Similar to the focus of our research, a study with German respondents examined the causal beliefs of people with an untreated mental illness and help-seeking intentions (23). The results of this study demonstrate that the intention to seek professional help is highest when the perceived cause is biomedical and is significantly lower for person-related causes, childhood trauma, stress, and unhealthy behaviors (23).

There is also some scientific research in the context of sleep-related complaints. Studies show that one third of the Canadian population and over 50 percent of the Italian population suffer from sleep complaints (2, 50). There is also evidence of an increase in prevalence during the pandemic (51). In general sleep-related complaints are positively correlated with aging (52), being female, having a low household income, lower education, and physical as well as psychological problems (53). However, only one out of ten of the affected use professional help (2). People significantly seek more professional help if they have higher education (54). The severity of the impairment also plays an important role here. People who report having daytime impairment due to their sleep complaints, tend to seek professional help more often than people without daytime impairment (54).

In the rare case that people seek professional help for their sleep complaints, they are often treated with prescription drugs (50, 55). In contrast to this, the image of the best possible “natural sleep” without any aids persists (56). Another way are self-help strategies. In a Chinese study 32% of the respondents with sleep-related complaints reported self-help strategies like relaxation and reading (57). Women are more likely to try self-help methods for dealing with their sleep-related complaints whereas men are more likely to use medication (58, 59).

Despite the extensive literature on this topic, we are unaware of any studies that have examined the influence of perceived cause on help-seeking behavior for sleep-related complaints. Such research is important to understand why and in which cases people tend to prefer to not seek professional help, although there are numerous health-related consequences for untreated sleep complaints. In the field of psychiatric disorders, illnesses like attention-deficit hyperactivity disorder (ADHD), depression, and bipolar disease are known to be related or accompanied by sleeping difficulties (60, 61). There are effects in both directions. Sleep complaints also affect psychiatric disorders. The relationship between these two groups is complex and bidirectional (62). It has also been shown that people who suffer from sleep-related complaints are prone to other comorbidities. An association has been found between people with sleep complaints and a higher rate of comorbidity with conditions such as anxiety-depressive disorders, other psychiatric disorders, chronic pain, and cardiovascular diseases (50, 63). Sleep deprivation also correlates with weight gain, increased risk of diabetes type 2, dementia, cognitive impairment and car accidents (8, 9, 64–66). For example, 24 h of sleep deprivation leads to cognitive impairment similar to a blood alcohol concentration of 100 mg/dL (67).

Research results point out the massive consequences of sleep-related complaints for the individual patient, for the society, and the healthcare system. This highlights the importance of examining sleep complaints and help-seeking behavior and especially an understanding of fundamental parameters that influence the help-seeking behavior like the perceived cause. If these parameters and causes are identified, organizational and structural adjustments may lead to better access and treatment of people with sleep complaints.

## 2 Materials and methods

### 2.1 Sample

The data basis for this study is the Healthcare-seeking in Germany (HEALSEE) survey (24). This representative survey provides detailed information on the decision-making behavior in the event of illness and the use of healthcare services by people aged between 40 and 75 in Germany. The sample is composed of 3,000 individuals who were selected through a multi-stage random process utilizing a dual-frame approach, whereby synthetic telephone numbers were generated. The computer-assisted telephone interviews (CATI) of the persons took place between April and August 2015. The focus of the study was on how people respond to and make decisions in the case of back pain, digestive complaints, and sleep complaints.

All participants were first asked whether they had back pain, digestive complaints, or sleep-related complaints in the past 3 months. If none of these applied, these individuals were classified as respondents with no symptoms. The exact question for recording sleep complaints was as follows: *“Have you had problems falling asleep, trouble sleeping through the night, or an excessive need to sleep at least once in the last three months?.”* This was followed by a measure of the frequency of the problem(s), with the response options *once*, *now and then,* and *more often/regularly*. In the case of the presence of more than one of the complaints mentioned, the classification was made based on what the respondents described as their most frequent symptom. If the frequency was also equal, the complaints referred to in the following survey was chosen at random. It should be noted that the data presented in this study are based solely on self-reports and did not include prior or further diagnostic procedures, such as clinical interviews or medical examinations. Participants were then asked specific questions about the symptom, how they deal with the symptom, health attitudes, health literacy, data on general health and their social network. Furthermore, socioeconomic characteristics were surveyed as well as institutional factors such as insurance status and distance to the next general practitioner. Since it is in our interest to investigate the utilization behavior of persons with sleep-related complaints, all persons belonging to the other symptom groups or to the group without symptoms are excluded from the analysis. The result is an initial sample size of 761 respondents.

### 2.2 Variables

This chapter provides a comprehensive account of the operationalization of the variables employed in the study. In addition to the dependent variable of the utilization of professional help and the perceived cause as the central predictor, the other explanatory factors are derived from the Behavioral Model of Health Services, which was presented in section 1.1.

#### 2.2.1 Utilization of healthcare services (DV)

The respondents were presented with a whole range of possible interventions and were asked which of these they had already taken in response to the complaints. In addition to low-threshold measures such as the use of a home remedy or an internet search, the surveyed interventions ranged all the way to physician visits. The last one is covered by the questions whether a family physician, a specialized physician, a hospital/emergency room, or a psychologist/psychotherapist was ever consulted due to the symptoms. The four binary variables were again combined into one dummy variable. As soon as a person has ever contacted or visited one or more of the four provider groups because of his or her sleep complaints, this is considered as seeking professional help.

#### 2.2.2 Cause of the symptoms (central IV)

The cause of the complaints serves as the central explanatory factor. The introductory question was as follows: *“To what cause(s) do you yourself attribute your sleep disturbances?.”* The respondents were given several possible causes and could also add additional reasons. The open statements were taken into account in the generation of the variable. First, however, these were checked to ensure that the information did not reflect one of the predefined categories. In this case, the information was post-coded. Among other things, it was asked whether mental problems are a possible cause. The exact wording of this question was as follows: “Do you attribute your sleep complaints to poor mental health, such as stress, worries or family problems?” In order to be able to check the extent to which people who suspect a mental cause for their complaints show a different utilization behavior, we created a variable with three values. It distinguishes between people who see only mental health problems as the cause, those who see mental health problems as one of two or more causes, and those who see one or more causes other than mental health. This offers the possibility to differentiate whether the mental aspect is seen as a partial or the sole reason.

#### 2.2.3 Need (symptom severity and duration)

As further important covariates, we consider the perceived daily impairment by the complaints and its duration. The severity of the symptoms is expressed on a five-point scale from “not at all” to “very much,” with intermediate gradations. When being asked for how long the symptoms have already persisted, it was possible to give an exact date. The dispersion of the resulting variable is highly right skewed, therefore we use the logarithm.

#### 2.2.4 Predisposing factors

We also control for general sociodemographic characteristics and health-related networks. We use education in the form of the highest school-leaving qualification, gender, migration background, and age. For age, the regression diagnostics showed that there is a curvilinear relationship. For this reason, the quadratic term of the age variable was also included. To adequately account for the presence of networks within healthcare, people were asked how easy it is for them to find someone who could recommend a physician. Originally, the variable had four values, but due to the small number of people for whom it is “not” or “not so easy” to get a recommendation, these two categories were combined. Also, a binary variable is included, which contains the information whether there is a psychologist and or doctor in the circle of family, friends, or acquaintances. To capture how people deal with health complaints as well as general problems, we consider several attitudinal questions. For the openness of dealing with symptoms, we take the number of people they have told about the complaints as an indicator. Furthermore, the competence of general practitioners, specialists, and psychologists with regard to one’s own complaints is provided. Since the dependent variable of utilization includes all three professions, a new variable was formed corresponding to the highest competency score in each case. A high score means that at least one profession is rated as competent to alleviate one’s own symptoms. In addition, on a four-point scale from “Do not agree at all” to “Agree completely,” the general handling of problems (in the following, deliberation) was recorded on the one hand and the handling of pain and other symptoms (in the following, self-determination) on the other hand. In the first case, a high level of agreement means that one first looks at a problem in detail before making a decision on how to deal with it. In the case of the second, agreement expresses that one first tries to always immediately want to know the cause of health problems.

#### 2.2.5 Enabling resources

To account for differences in utilization due to a lower coverage rate, the distance to the primary care physician as well as to the nearest hospital is added. Another institutional factor considered is the type of health insurance (statutory health insurance or private health insurance). For instance, patients with private health insurance have a shorter average waiting time for specialist appointments (68). A detailed overview of the descriptive statistics for the variables is provided in Table 1.

**Table 1:** Descriptive statistics of the variables.

Variables	Number of cases	Mean/relative frequency (italic values)
Utilization of professional healthcare services		
No Utilization	294	*47.57*
Utilization	324	*52.43*
Assumed cause		
Not mental	186	*30.10*
Partial mental	256	*41.42*
Exclusively mental	176	*28.48*
Need		
Symptom severity (Min. = 1; Max. = 5)	618	2.79
Log. duration of symptoms in years (Min. = −4,80; Max. = 4,09)	618	1.48
Predisposing factors		
Education		
Low	117	*18.93*
Medium	235	*38.03*
High	266	*43.04*
Sex		
Male	249	*40.29*
Female	369	*59.71*
Age in years (Min. = 40; Max. = 75)	618	56.46
Migration background		
No Migration background	565	*91.42*
Migration background	53	*8.58*
Recommendations for physicians		
Not at all/not so easy	81	*13.11*
Rather easy	213	*34.47*
Very easy	324	*52.43*
Physicians/psychologist among acquaintances		
No	300	*48.54*
Yes	318	*51.46*
Openness (Min. = 1; Max. = 6)	618	2.95
Competence of physicians/psychologists		
Not at all/poorly	123	*19.90*
Rather	296	*47.90*
Very	199	*32.20*
Self-determination (Min. = 1; Max. = 4)	618	3.33
Deliberation (Min. = 1; Max. = 4)	618	3.20
Curiosity (Min. = 1; Max. = 4)	618	3.32
Enabling resources		
Type of health insurance		
Statutory health insurance	505	*81.72*
Private health insurance	113	*18.28*
Distance family physician (Min. = 0; Max. = 60)	618	7.79
Distance hospital (Min. = 1; Max. = 90)	618	16.95

Data basis HEALSEE (24), own calculations. Range in parentheses for metric variables. *N* = 618.

### 2.3 Analytical strategy

Due to the nature of the dependent variable, a logistic regression is performed as follows. A total of three models are estimated, first a simple model with utilization as the dependent variable as well as the type of cause, the severity, and the duration of the symptoms (need). The second model also takes predisposing factors such as sociodemographics, personal health network and attitudes into account. The last model also includes enabling resources. In this way, it can be ensured whether the suspected correlation is still evident when controlling for already known influencing factors. Moreover, an additional logistic regression model was calculated for all individuals who indicated that they had never sought professional help for their sleep complaints. This model included medication use as the dependent variable. The specific question posed was, “Have you ever taken or used any medication to alleviate your sleep complaints?” The same variables were used as explanatory factors as in the final model for utilization. Instead of odds ratios, Average Marginal Effects (AME) are reported as this allows easier interpretation and better comparability of the coefficients between tested models (69). In this particular context, the AMEs illustrate the average change in the probability of utilization in relation to an increase of one unit in the respective independent variable. Due to the large number of variables used, there are observations that have to be excluded due to missing values. Of the 761, a total of 618 (81%) persons have valid values for all variables. All analyses were performed using STATA 18 (StataCorp LLC).

## 3 Results

**Figure 1:**
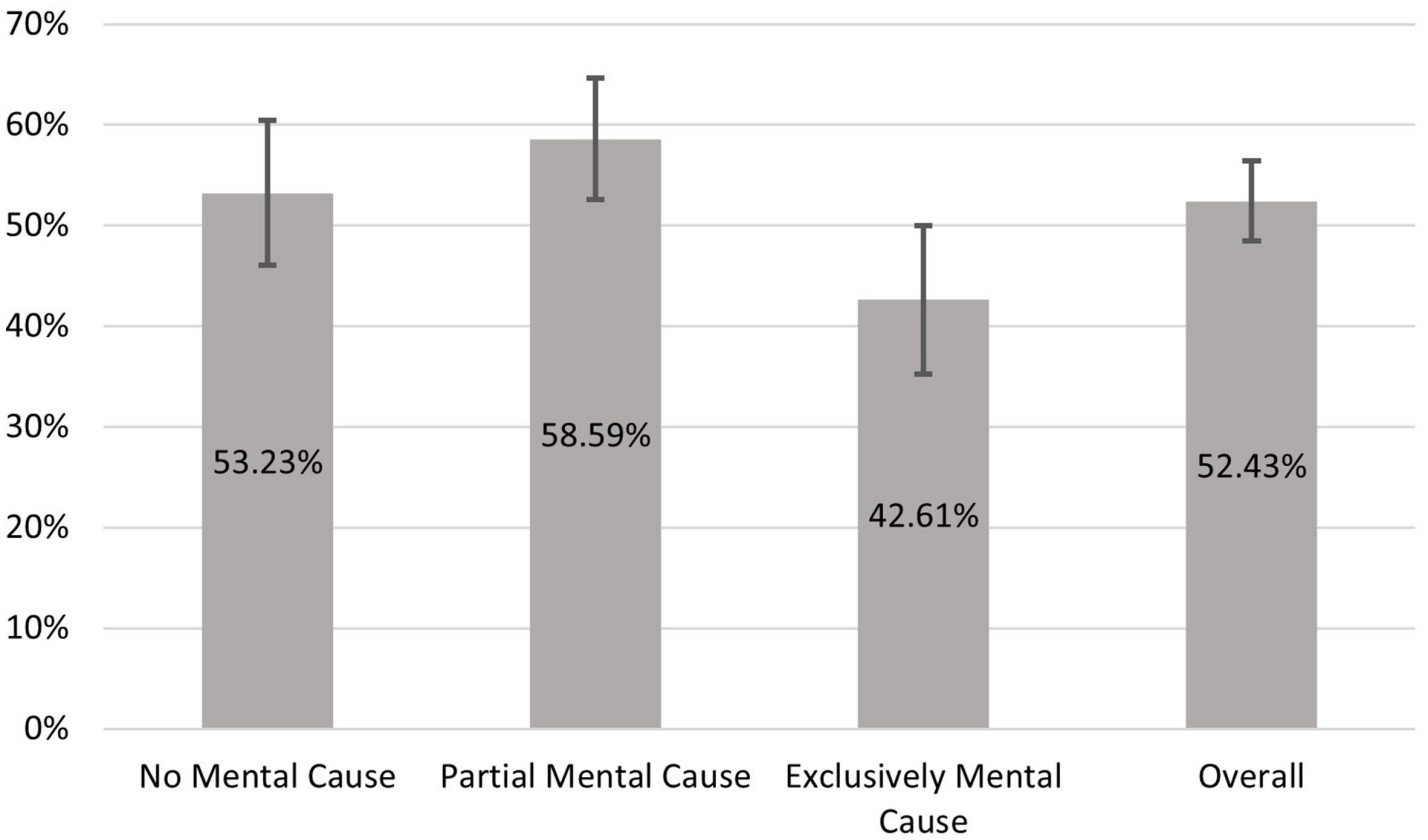
Utilization depending on the assumed cause of the symptoms. Data basis HEALSEE (24), own calculations. *N* = 618.

We begin with an overview of the dependent as well as the central independent variable. Figure 1 shows the level of use of professional help depending on the suspected cause. With about 52% (95% CI = 48.48 to 56.38), more than half of the respondents have consulted professional help for their sleep complaints. About 48% had not yet consulted professional help. There are considerable differences depending on the suspected cause. People who do not assume that their mental health is involved have used professional help slightly more frequently than average (53%; 95% CI = 46.02 to 60.43). The percentage is even higher (58%; 95% CI = 52.54 to 64.65) among those who assume that psychological problems play a role but that there are other causes as well. There is a large difference of 10, respectively, 16 percentage points compared with people with an exclusively mental cause (43%; 95% CI = 35.27 to 49.95). The considerable mean differences between this group and the other two groups are also statistically significant. With regard to the research question, this first simple comparison of mean values already shows clear differences depending on the suspected cause. As anticipated, the group that suspects an exclusively mental cause reported the lowest level of utilization.

The results of the multiple logistic regression illustrate the extent to which these differences persist or change when controlling for other variables (see Table 2). The first model includes, in addition to the cause, the symptom severity and duration as covariates. Even controlling for these two additional factors, there is a significant difference between the reference category (exclusively mental) and not or partial mental. However, the largest difference from the reference category is not partial mental, as in the descriptive statistics, but not mental. More specifically, the probability of utilization is approximately 16% higher when it is assumed that no mental component is involved (AME: 0.158; *p* < 0.01). Furthermore, as expected, the probability of seeking help increases with increasing impairment (AME: 0.171; *p* < 0.001). The same applies to the duration (AME: 0.050; *p* < 0.001). The longer a person has complaints, the more likely it is that he or she is consulting professional help.

**Table 2:** Results logistic regression.

Variables	AME	S.E.	AME	S.E.	AME.	S.E.
Assumed cause (exclusively mental)						
Not mental	0.158**	0.047	0.117**	0.044	0.117**	0.044
Partial mental	0.106*	0.044	0.094*	0.040	0.093*	0.040
Need						
Symptom severity	0.171***	0.014	0.120***	0.015	0.122***	0.015
Log. duration of symptoms	0.050***	0.012	0.041***	0.012	0.041***	0.012
Predisposing factors						
Education (low)						
Middle			0.001	0.049	−0.005	0.049
High			−0.032	0.050	−0.047	0.051
Sex (Male)						
Female			0.012	0.035	0.014	0.035
Age			0.072**	0.022	0.071**	0.022
Age squared			−0.001**	<0.001	−0.001**	<0.001
Migration background (None)						
Migration background			0.162*	0.060	0.166**	0.059
Recommendations for physicians (not at all/not so easy)						
Rather easy			−0.124*	0.055	−0.133*	0.055
Very easy			−0.106	0.053	−0.120*	0.054
Physicians/psychologist among acquaintances. (No)						
Yes			−0.052	0.034	−0.054	0.034
Openness			0.070***	0.008	0.071***	0.008
Competence of physicians/psychologists (not at all/poorly)						
Rather			0.063	0.045	0.060	0.045
Very			0.094	0.048	0.094	0.048
Self-determination			−0.052*	0.023	−0.050*	0.024
Deliberation			0.045	0.023	0.045	0.023
Curiosity			0.043*	0.021	0.042*	0.021
Enabling resources						
Type of health insurance (statutory health insurance)						
Private health insurance					0.045	0.044
Distance family physician					−0.001	0.003
Distance hospital					−0.001	0.002
Adj. *R*^2^_MF_	0.14		0.22		0.22	

Data basis HEALSEE (24), own calculations. *N* = 618. Reference categories in parentheses *p < 0.05, **p < 0.01, ***p < 0.001.

The second model also takes into account socio-demographic variables as well as relevant network and attitudinal variables of the individuals. The difference between an exclusively psychological cause and the other manifestations nevertheless remains stable and continues to be significant (Not mental - AME: 0.117; *p* < 0.01; Partial Mental - AME: 0.106; *p* < 0.05). The same applies for the duration and severity of the complaints. Of the sociodemographic variables, age and migration background are statistically relevant. Persons with a migration background have a significantly higher utilization level. For age, there is a curvilinear relationship. This means that with increasing age, the probability of utilization initially increases until this effect weakens and decreases again at an advanced age. Of the other predictors included in the second model, openness in dealing with sleep-related complaints in particular contributes to explaining the different utilization behavior. The more openly and with the more people one talks about it, the more likely one is to use professional help. Always wanting to know the exact cause as soon as one is confronted with health complaints also leads to an increase, whereas high self-determination seems to be an inhibitor. A network of contacts in the healthcare system is not conducive to seeking professional healthcare for sleep-related complaints, at least when other variables are taken into account. The possibility to get recommendations for doctors without problems even has a significant negative effect.

In the third model, we additionally control for the institutional characteristics of the respondents. The addition changes the previous effects only marginally. Moreover, none of them has a significant impact on the dependent variable. Thus, the institutional factors used in our analysis seem to play a minor role. In consideration of the research question, it can be stated that even when known factors are taken into account, the perceived cause also contributes significantly to the explanation of help-seeking behavior for sleep complaints.

**Figure 2:**
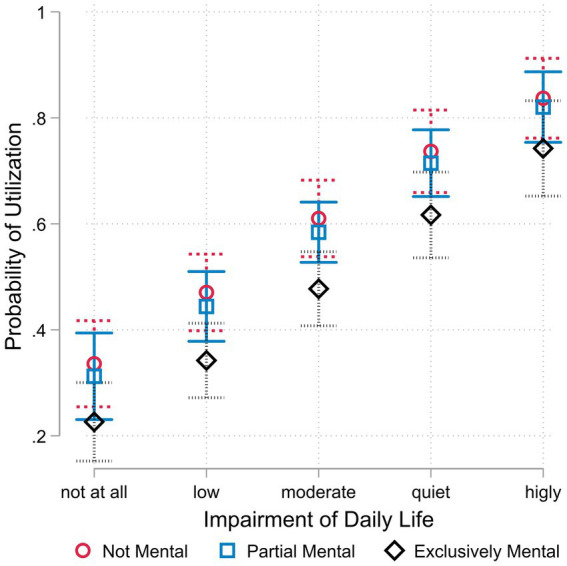
Predicted probability of utilization by cause and severity of symptoms. Data basis HEALSEE (24), own calculations. *N* = 618; 95%- Confidence intervals.

In order to gain a more accurate understanding of the extent to which there is a genuine risk of underuse, we examine whether the discrepancy in seeking professional assistance is also evident in cases of severe complaints or whether the observed effect is largely attributable to the reluctance of individuals with only mild complaints to seek help if they suspect a psychological cause. Figure 2 displays the predicted probabilities for the three cause groups for all five degrees of impairment. For all severity levels, the probability of utilization is highest for those without any mental health involvement. For those with partial involvement, the values are only minimally lower and never significantly different. For people with an exclusively psychological cause, however, the predicted values are significantly lower. Although the magnitude of the differences decreases somewhat with increasing impairment, it does so only slowly. For example, the probability of utilization in the case of a moderate impairment of everyday life due to the complaints for people with no or only partial involvement of their mental health is still around 60% and that of those with exclusively mental health problems is 47%. In the case of very severe complaints, the differences become smaller and the difference between a partial mental involvement and an exclusively mental cause can no longer be statistically confirmed on the highest level of complaints. However, even then there is a difference of almost 10 percentage points. In addition, the other values differ significantly from each other, even if the symptoms have a strong negative effect on coping with everyday life.

**Figure 3:**
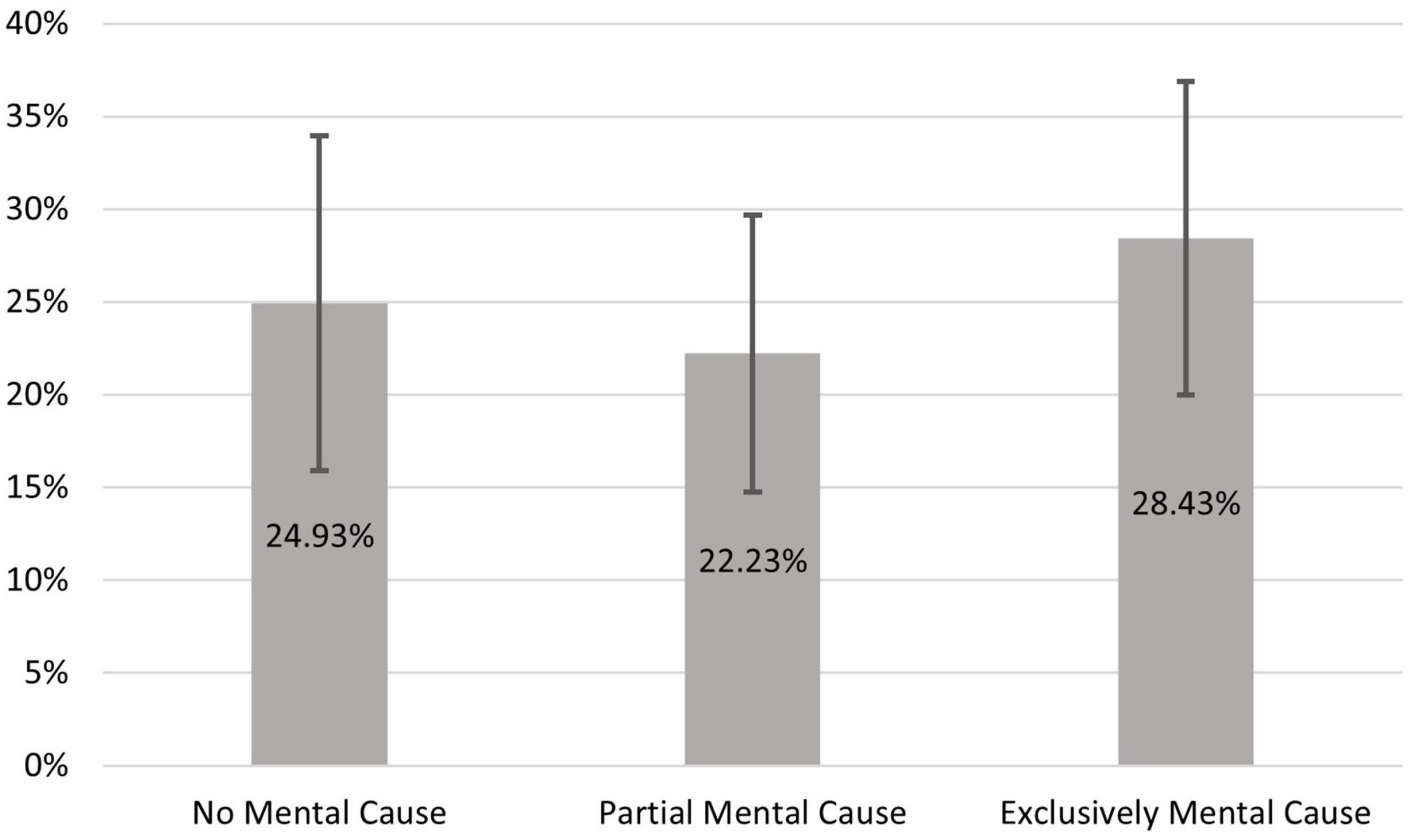
Predicted probability of medication use of individuals who did not seek professional help. Data basis HEALSEE (24), own calculations. *N* = 275; 95%- confidence intervals.

Our findings indicate a risk of undertreatment of sleep complaints in individuals who consider their mental health to be causal. The fact that the differences in utilization persist even with very severe complaints once again underlines the problem. However, it is still unclear to what extent self-medication may be occurring in addition to the risk of underuse. The results of the logistic regression, with the intake of medication as the dependent variable, are presented in Figure 3. It can be observed that individuals who hypothesize an exclusively psychological cause are the most likely, at approximately 28.5% (95% CI = 19.96 to 36.89), to have previously taken medication for their symptoms. For those without a psychological cause, the share is less than a quarter (95% CI = 15.92 to 33.93), and for those with a partial cause, it is only 22% (95% CI = 14.75 to 29.70). In addition to underuse, there also appears to be a risk of medication misuse for people with sleep problems, especially if they suspect a mental cause. It should be noted, however, that the differences are not significant and the confidence intervals are quite large. One reason for this is the small number of cases (*N* = 275) for this model.

## 4 Discussion

The objective of this analysis was to examine whether there is a difference in help-seeking behavior for sleep-related complaints if the underlying cause is assumed to be psychological. First of all, the results show that the help-seeking behavior for sleep complaints is slightly higher than 50%, regardless of the assumed cause. This finding is consistent with previous results (54), although there are also studies that have identified significantly lower utilization rates (2). One potential explanation for this discrepancy is the use of disparate operational definitions and country-specific factors. In relation to the research question, the study provides significant results. While individuals who perceive a non-mental or only partially mental cause for their sleep complaints are more likely to engage in higher levels of help-seeking behavior, there is a significantly lower use of health services for sleep disorders due to mental reasons. This underlines the results of previous studies which have shown that the use of professional help for mental health problems is often quite low (16, 17). One crucial factor contributing to this phenomenon is the stigmatization of mental illnesses. As proven in several studies, stigmatization is a major barrier to healthcare utilization (19–21). This problem, including medical gaslighting of mental health issues, is particularly serious for certain population groups such as women and minorities (70). If patients experience stigmatization and as a consequence remain untreated, the result could be underutilization of necessary treatment (71). The findings of our study indicate that these discrepancies in help-seeking behavior were evident even in cases of severe symptoms. Furthermore, there were indications that people who perceive the cause as psychological are the individuals who increasingly take medication on their own. Self-medication might be a risky method for dealing with sleep complaints, because there is an unknown efficacy and safety of herbal and non-prescription medications (72). This leads us to conclude that there is a need for measures that encourage people to seek professional help for sleep problems. It is important to resolve this discrepancy between having sleep complaints and dealing with it professionally, especially in view of the personal impairment, health risks and possible co-morbidities (50, 63).

### 4.1 Policy recommendations

We suggest the implementation of measures that remove the personal barriers that are felt through the ongoing stigmatization of mental health issues within society. Stigma results in the alteration of a person’s image simply by virtue of a characteristic such as mental illness (37). Presenting a holistic picture of a person in the media can lead to destigmatization, as not just the characteristic effects of the mental health illness is drawn (73). In addition to destigmatization, improving mental health literacy is another important factor in removing personal barriers to seeking help for mental health problems. Knowing about the symptoms, the treatment and help-seeking sources can improve the possibility for help-seeking (74).

Not only is it important to find a solution to the gap in help-seeking behavior due to the perceived cause, but also to find a way to engage and collect those people who have not yet taken the next step to professional help. This is particularly important in view of the fact that longer working hours make it more difficult for women to utilize health services, which could make the situation even more problematic in the future (75). There are two levels that should be addressed in regard to resolve the discrepancy between sleep-related complaints and dealing with it professionally. The first is the help seeking behavior itself. Although there are predictors which increase the probability to seek professional help such as severity and duration, openness and age, the access to professional help needs to be made more low-threshold. Cognitive-behavioral therapy (CBT-I) and cognitive-behavioral self-help therapy (Digital CBT-I) are treatment methods, which fulfill these requirements. (d)CBTI is recommended as first-line treatment of sleep complaints by the American College of Physicians and has been adopted by European countries. The efficacy of this approach has been validated, and it can achieve relatively quick results (76, 77). Both CBT-I and dCBT-I consist of different strategies as sleep restriction, stimulus control and cognitive reappraisal (78). In contrary to CBT-I which is provided through face-to-face individual or group treatment, dCBT-I is the digital version of this program and entirely self-directed. While CBT-I has indicated superior outcomes, dCBT-I is also beneficial for sleep complaints (79). Studies illustrate that CBT-I is as effective as sedative medication during acute treatment of 4–8 weeks (80) and is more effective in the long run (81). Positive experiences with dCBT-I could lead people to a more open mindset about mental health and encourage them to seek further psychological help. We further suggest that conversations should be initiated by the physician in order to shift the burden of individual responsibility of the patient to the systematic assessments by healthcare professionals. When seeing physicians, patients often state physical pain, but as physicians become more aware of impairments like sleep complaints and their side effects, the physicians should proactively address the topic themselves (55). This additional engagement by physicians would mean an increase in time, effort and consequently costs, but in the long-term this may lead to a decrease in the high costs for the economy and society (10, 82, 83).

The second level is to address the society. As long as sleep complaints are not regarded as an important health problem, people will not take them seriously. We advocate for a common awareness of the illness and its consequences (64, 66). Placing this information in media, such as social media, news, films or series, where it can be accessed by almost everyone, can be a way of raising awareness (84). Given that general practice surgeries and treatment centers are the initial point of contact for individuals experiencing health-related issues, they can serve as a conduit for disseminating informational leaflets pertaining to specific symptoms and potential treatment options for sleep-related complaints.

After all, it is not just one thing that needs to be changed, but several measures at different levels that need to be addressed in order to increase help-seeking behavior not only for mental but also for partially mental and non-psychological causes.

### 4.2 Limitations

It is evident that the data set, while representative, is limited in that it refers only to individuals from Germany between the ages of 40 and 75 years. Therefore, it is not possible to transfer the results in a one-to-one manner to other countries or age groups. In addition, the analyzed data are cross-sectional, which, in contrast to panel data, precludes the possibility of inferring causal effects. Moreover, the data set exclusively contains information on individuals who had registered complaints within the three-month period preceding the survey. While this approach excludes individuals with complaints from longer ago, it mitigates the potential for recall bias. It is also crucial to highlight that no clinical interviews or other medical examinations were conducted to verify the reported symptoms. Instead, the data was based solely on self-reports made via telephone. One of the study’s key strengths is its comprehensive consideration of the numerous factors that influence help-seeking behavior, which reduces the risk of obtaining distorted results due to a lack of consideration. It must be acknowledged, however, that the model cannot be considered complete.

## 5 Conclusion

More and more people are affected by mental health problems (85). This is a growing societal and economic problem as indicated by the number of working days lost due to mental illness that in Germany increased by 56% between 2010 and 2020 (85). Sleep complaints and related mental health problems are a growing burden for individuals, the economy, and the society as a whole. Our study suggests that stigmatization plays a key role in utilization. Therefore, better solutions on how to identify and solve sleep complaints are highly required. Further research should therefore focus more on the possible stigmatization and expand existing explanatory models of utilization behavior to include this factor. This may, in turn, facilitate the identification and reduction of potential instances of underuse and misuse of medication. In addition, however, it should also be investigated what the treatment looks like when professional help is sought and whether the approach is adequate.

## Data Availability

The datasets presented in this study can be found in online repositories. The names of the repository/repositories and accession number(s) can be found below: https://search.gesis.org/research_data/ZA5248.
